# Genetic analysis of ‘*Candidatus* Phytoplasma aurantifolia’ associated with witches’ broom on acid lime trees

**DOI:** 10.7717/peerj.4480

**Published:** 2018-03-05

**Authors:** Aisha G. Al-Ghaithi, Ali M. Al-Subhi, Issa H. Al-Mahmooli, Abdullah M. Al-Sadi

**Affiliations:** Department of Crop Sciences, College of Agricultural and Marine Sciences, Sultan Qaboos University, Al-Khod, Muscat, Oman

**Keywords:** WBDL, Acid lime, Oman, Phylogeny

## Abstract

“*Candidatus* Phytoplasma aurantifolia” is associated with witches’ broom disease of lime in Oman and the UAE. A previous study showed that an infection by phytoplasma may not necessarily result in the physical appearance of witches’ broom symptoms in some locations in Oman and the UAE. This study investigated whether phytoplasma strains belonging to “*Ca.* P. aurantifolia” (based on the 16S rRNA gene analysis) in locations where disease symptoms are expressed are different from phytoplasma in locations where disease symptoms are not expressed. About 21 phytoplasma strains (15 from areas and trees with disease symptoms and six from areas and trees without disease symptoms) were included in the analysis. The study utilized sequences of the imp and SAP11 genes to characterize the 21 strains. Phylogenetic analysis of both genes showed that the 21 strains are similar to each other and to reference strains in GenBank. The study shows that there is a low level of diversity among all phytoplasma strains. In addition, it shows that phytoplasma in places where witches’ broom symptoms are not expressed are similar to phytoplasma in places where disease symptoms are expressed. This may suggest that disease expression is not linked to the presence of different phytoplasma strains, but may be due to other factors such as weather conditions.

## Introduction

Phytoplasmas are prokaryotic gram-positive bacteria that are difficult to be cultured in artificial media (Contaldo et al., 2012, 2016). They are phloem limited and transmitted by phloem-sucking insects of the order Hemiptera, mostly leafhoppers (*Cicadellidae*), planthoppers (*Fulgoroidea*), and psyllids (*Psyllidae*) (Frost et al., 2013; Rashidi et al., 2014; Queiroz et al., 2016). They have a wide range of host plants from over 100 plant families, including many citrus species (Hogenhout et al., 2008).

Symptoms produced by “*Candidatus* Phytoplasma aurantifolia” on acid lime trees include excessive production of shoots with very small, light green leaves and short internodes, no flowers or fruits, and the general decline of the tree leading to a final dieback. Witches’ broom symptoms progress rapidly from the time of symptom appearance until the final stage of tree death where the tree collapses within four to five years after first symptom appearance (Al-Sadi et al., 2012; Al-Yahyai et al., 2015).

A previous study showed that the association of “*Ca*. P. aurantifolia” with acid lime results in witches’ broom symptoms in some geographical locations but not in others (Al-Ghaithi et al., 2017). The study found the symptoms were apparent in most areas in Oman except in monsoon areas or areas with extreme desert conditions. Although this may be in part related to differences in climatic conditions between these areas, it is necessary to characterize the phylogenetic association and diversity of phytoplasma that are present in locations which are conducive and less conducive to witches’ broom symptom expression.

The identification and characterization of phytoplasma has long relied on the use of the 16S rRNA gene (Mehdi et al., 2012). However, other molecular markers have been developed which include the *Tuf*, *SecA, SecY, SAP*11, *imp,* and other genes (Sugio et al., 2011; Bekele et al., 2011; Dickinson & Hodgetts, 2013; Al-Abadi et al., 2016). Immunodominant membrane proteins (*imp*) genes are more variable than their surrounding genes in phytoplasma. They are located on the external surface of phytoplasma cell membrane (Siampour et al., 2012). SAP11 is an effector protein that targets plant cell nuclei (Bai et al., 2009) and induces stem proliferation, changes in leaf shape, and the down regulation of jasmonic acid production.

Our previous work compared phytoplasma across countries (Al-Abadi et al., 2016) using three genes but only from one area (subtropical). In addition, a recent study by our group compared phytoplasma across different conditions using one gene (16S rRNA) but from three areas (semitropical, subtropical, and desert) (Al-Ghaithi et al., 2017). The aim of our current study was to investigate the genetic relatedness of phytoplasma across different conditions (semitropical, subtropical, and desert) using two additional genes (*imp* and SAP11) that were not used in 2017.

The study helped to determine whether the difference in witches’ broom symptom expression in different geographical regions could be related to the presence of different phytoplasma strains.

## Materials and Methods

### Sample collection

**Figure 1:**
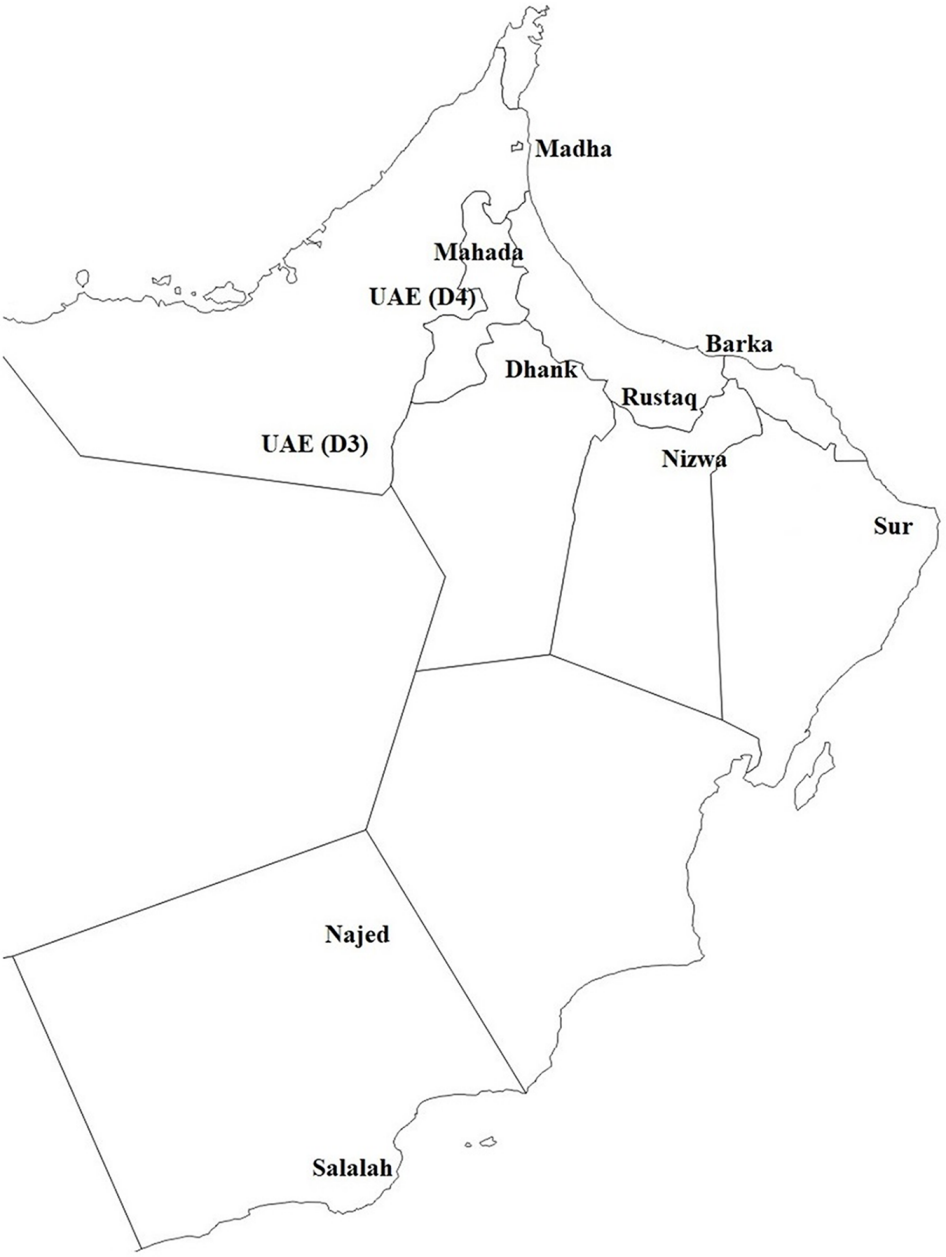
A map showing the locations from which samples were collected. See Table 2 for more details.

Fifteen samples positive for phytoplasma from trees developing WBD symptoms and six samples collected from asymptomatic trees from areas with no apparent WBD symptoms were included in the study. The choice of samples was based on our previous findings (Al-Ghaithi et al., 2017). The 21 samples were collected from 11 different locations, two samples from a semitropical area (Salalah), four samples from desert areas (Najed and UAE), and 15 samples from subtropical areas (Fig. 1). Samples were collected from young leaves developing WBD symptoms.

### Nucleic acid extraction and polymerase chain reaction

Total nucleic acids were extracted following the method of Doyle & Doyle (1987) in CTAB extraction buffer. Ground leaves mixed with CTAB buffer were incubated at 65 °C for 10 min. This was followed by adding an equal amount of phenol:chloroform:isoamyl alcohol (25:24:1) and centrifugation (this step was repeated twice). Then 0.6 volume of isopropanol and 0.3M NaAc (pH 5.2) was added to the supernatant. The DNA pellets were washed with 70% ethanol, dried and then resuspended in 100 μl sterilized distilled water and stored at −80 °C.

The 21 strains have already been characterized in our previous study based on the 16S rRNA (Table 1) and were found to belong to the 16S rRNA subgroup II-B (Al-Ghaithi et al., 2017). Therefore, polymerase chain reaction (PCR) were conducted for the *imp* and *SAP11* genes as described by Al-Subhi et al. (2017). Imp-R1, Imp-F1, and Imp-F2 primers were used for the amplification of the *imp* gene using a two-stage PCR. Imp (F1) and Imp (R1) were used in direct PCR, while Imp (F2)/Imp (R1) were used in seminested PCR (Table 1). Amplification of the SAP11 gene was done using the primers SAP11-W-F1 and SAP11-W-R1 in direct PCR. The primer pair SAP11-W-F2/SAP11-W-R2 was used in nested PCR (Table 1). The PCR conditions for both genes in the direct and nested/seminested PCR were adjusted to the same conditions as follows: 94 °C for 2 min, then 40 cycles (94 °C for 30 s, 53 °C for 45 s, and 72 °C for 1:30 s), followed with final extension at 72 °C for 10 min.

**Table 1:** Primers used for amplifying Phytoplasma genes.

Gene	Primer name	5′-3′ Sequence	Product size (bp)	Reference
imp	imp-F	GTTATAATTGAAGGCGATA-TTG	519	Al-Subhi et al. (2017)
	imp-F2	ATAGAGGAGAAGAAAAAGTTTC		
	imp-R	GATCATATTTGGTTTATAGGAG		
SAP11	SAP11-w-F1	CTTCAGCCACAAATAGAATCTTT	1,050	Al-Subhi et al. (2017)
	SAP11-w-R1	CAAATACAAATCGCTGCATAAA		
	SAP11-w-F2	TTCCTTTTATGAAATCACCTCAG	∼550	
	SAP11-w-R2	GCGCATATTATTAAACTCCTTT		

**Note:**

Al-Subhi et al. (2017).

### Sequencing and analysis of sequences

Sequencing was carried out at MACROGEN Inc., Korea, using the same primers used in the nested PCR. The forward and reverse sequences for each phytoplasma isolate were aligned and edited using ChromasPro (v. 1.41; Technelysium Pty Ltd., Brisbane, Queensland, Australia). Phylogenetic analysis of the obtained sequences and representative sequences from GenBank for the different subgroups of phytoplasma was carried out using the Kimura 2-parameter evolutionary model in Mega 6 (Tamura et al., 2013). Trees were generated using 1,000 replications and a 50% majority-rule for the bootstrap analysis.

## Results

### Phylogenetic analysis of phytoplasma strains based on SAP11 gene

**Figure 2:**
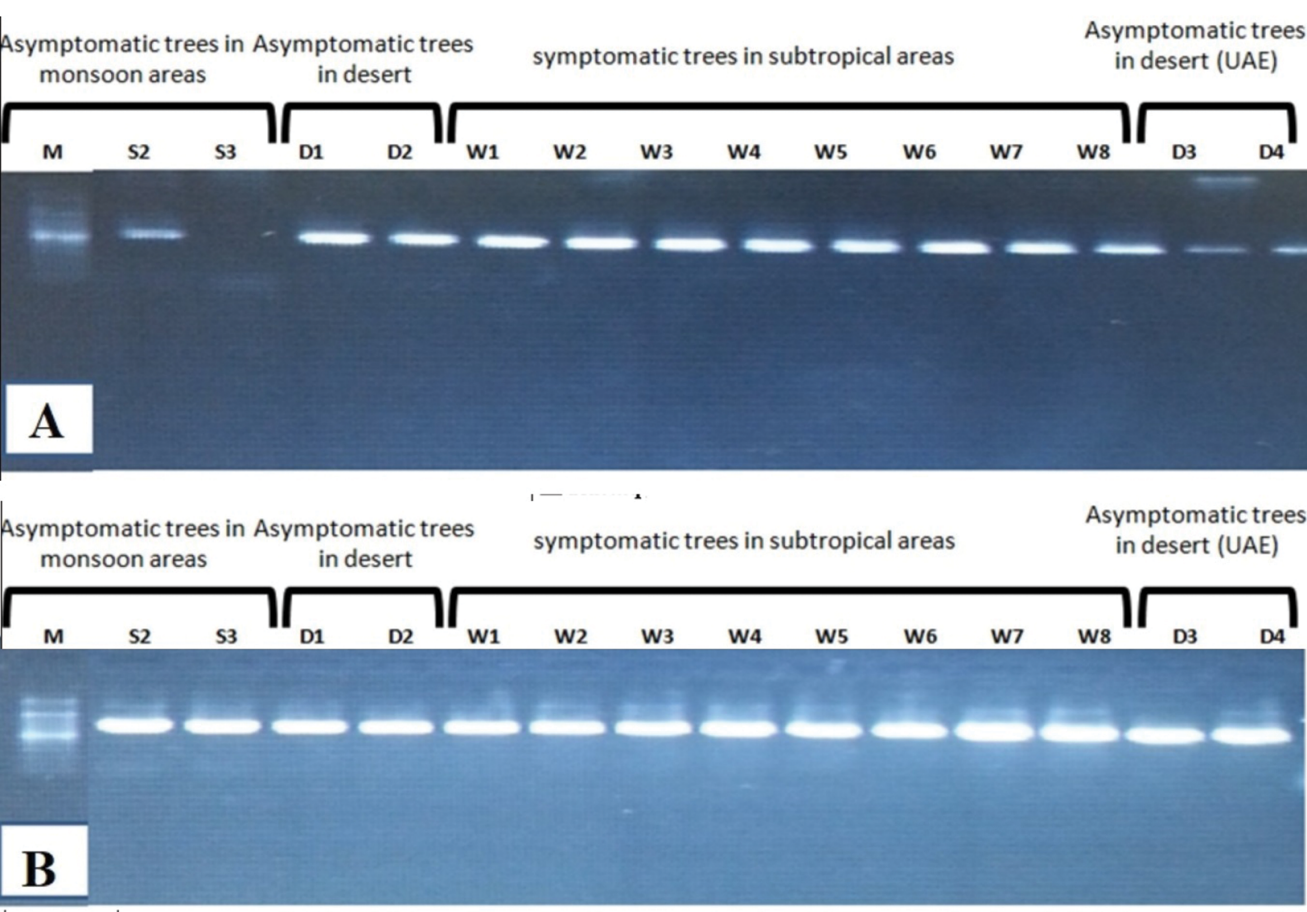
(A) Nested PCR of SAP11 gene using SAP11 F2/R2 showing 339 bp DNA fragment size. (B) Seminested PCR of IMP gene using IMP F2/R1 showing 519 bp for some samples from monsoon areas (semitropical), desert, and subtropical areas.

**Figure 3:**
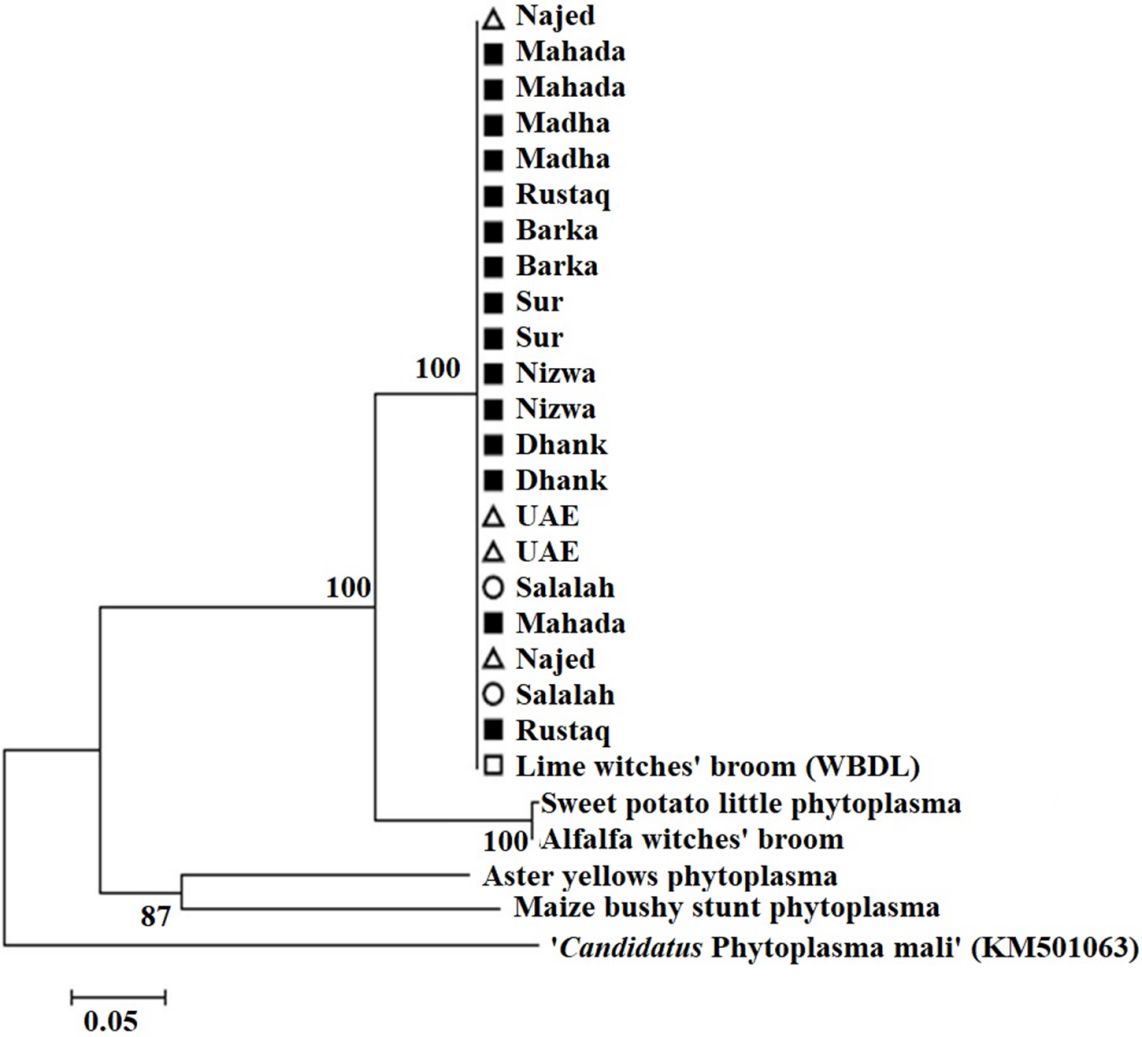
Phylogenetic analysis using the SAP11 gene of phytoplasma strains sampled in acid lime trees grown in desert areas (circle), semitropical areas (triangle), and subtropical areas (square). The tree was constructed by the neighbor-joining method using Kimura’s two-parameter mode. Sequences of the 21 strains from Oman were compared to a worldwide collection of strain sequences from different phytoplasma groups obtained from GenBank.

Amplification using the primer pair SAP11-w-F2/and SAP11-w-R2 produced a fragment of 339 bp (Fig. 2). Sequencing of the PCR products followed by phylogenetic analysis based on SAP11 gene sequences showed that all samples clustered together with the reference isolates belonging to “*Ca*. P. aurantifolia” (Fig. 3). All strains from Oman clustered with the reference isolate with a very high bootstrap support (100%), and were separated from all other closely related strains.

### Phylogenetic analysis of phytoplasma strains based on the *imp* gene

**Figure 4:**
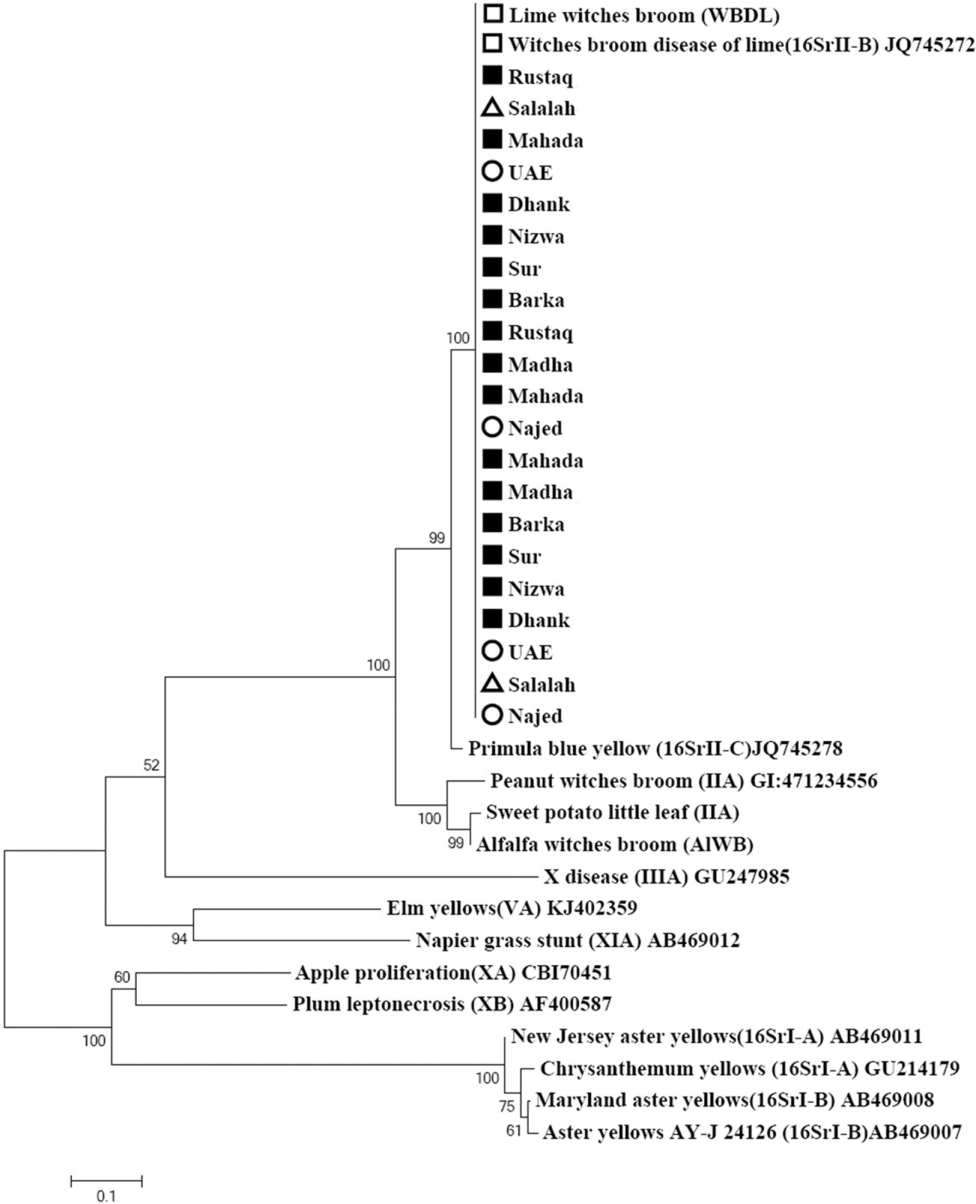
Phylogenetic analysis using the *imp* gene of phytoplasma strains sampled in acid lime trees grown in desert areas (circle), semitropical areas (triangle), and subtropical areas (square). The tree was constructed by the neighbor-joining method using Kimura’s two-parameter mode. Sequences of the 19 strains from Oman were compared to a worldwide collection of strain sequences from different phytoplasma groups obtained from GenBank.

The primer pair ImpF2/Imp R1 resulted in a product of 519 bp in size (Fig. 2). Phylogenetic analysis of the 21 samples and three reference sequences from GenBank showed that all samples clustered with the references strains with a very high bootstrap support (100%) (Fig. 4).

**Figure 5:**
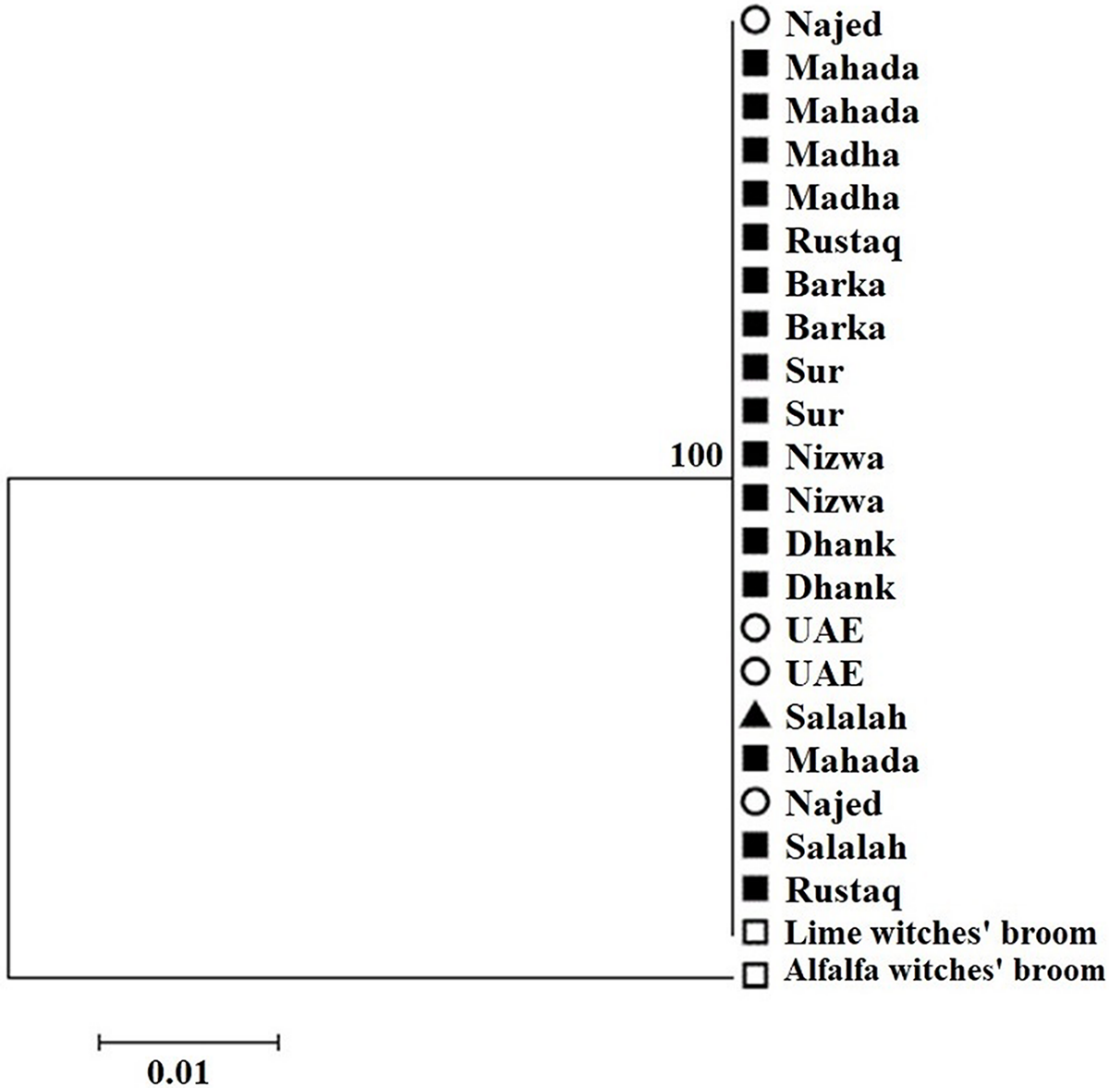
Phylogenetic analysis of 21 isolates sampled in acid lime trees grown in desert areas (circle), semitropical areas (triangle), and subtropical areas (square) based on the combined Imp and SAP11 gene sequences.

Phylogenetic analysis of 21 isolates based on the combined *Imp* and *SAP11* gene sequences showed clustering of all the isolates in one cluster (Fig. 5). GenBank accession numbers for all samples are illustrated in Table 2.

**Table 2:** GenBank accession numbers and characteristics of 21 phytoplasma isolates used in the study.

No.	Sample code	Name of the region	Environment	Year of collection	GenBank accession numbers		
					16S rRNA	imp	SAP11
1	S2	Salalah	Semitropical	2014	KX602312	KY829473	KY829493
2	S3	Salalah	Semitropical	2014	KX602309	KY829474	KY829494
3	D1	Najed	Desert	2014	KX602311	KY829475	KY829495
4	D2	Najed	Desert	2014	KX602290	KY829476	KY829496
5	D3	UAE	Desert	2015	KX602307	KY829477	KY829497
6	D4	UAE	Desert	2015	KX602308	KY829478	KY829498
7	W1	Mahada	Subtropical	2014	KX602293	KY829479	KY829499
8	W2	Mahada	Subtropical	2014	KX602294	KY829480	KY829500
9	W3	Mahada	Subtropical	2014	KX602310	KY829481	KY829501
10	W4	Madha	Subtropical	2014	KX602295	KY829482	KY829502
11	W5	Madha	Subtropical	2014	KX602296	KY829483	KY829503
12	W6	Rustaq	Subtropical	2014	KX602298	KY829484	KY829504
13	W7	Rustaq	Subtropical	2014	KX602313	KY829485	KY829505
14	W8	Barka	Subtropical	2014	KX602299	KY829486	KY829506
15	W9	Barka	Subtropical	2014	KX602300	KY829487	KY829507
16	W10	Sur	Subtropical	2014	KX602301	KY829488	KY829508
17	W11	Sur	Subtropical	2014	KX602302	KY829489	KY829509
18	W12	Nizwa	Subtropical	2014	KX602303	KY829490	KY829510
19	W13	Nizwa	Subtropical	2014	KX602304	KY829491	KY829511
20	W14	Dhank	Subtropical	2014	KX602305	KY829492	KY829512
21	W15	Dhank	Subtropical	2014	KX602306	KY829473	KY829513

## Discussion

Phytoplasmas of the taxonomic group 16SrII (peanut witches’ broom phytoplasma group) are associated with diseases affecting crops and wild plants in different geographical areas worldwide. “*Ca.* P. aurantifolia,” from the taxonomic subgroup 16SrII-B, causes a devastating and lethal disease of lime (Lime witches’ broom) in Gulf countries (Oman, UAE, and Saudi Arabia). Phytoplasma inducing a similar witches’ broom disease were reported in different host plants such as alfalfa (Khan et al., 2002) and sesame (Al-Sakeiti et al., 2005). Furthermore, the 16Sr II group was detected in other crops in the Middle East including Iran and Lebanon (Weintraub & Jones, 2010), the Mediterranean region (Tolu et al., 2006), Australia (Aryamanesh et al., 2011), Mexico (Hernandez-Perez et al., 2009), Indonesia (Harling et al., 2009), Europe (Tolu et al., 2006; Davino et al., 2007; Parrella et al., 2008; Harling et al., 2009), and Sudan (Zamora, Acosta & Martínez, 2012).

“*Candidatus* Phytoplasma aurantifolia” was reported in Oman, the UAE, Iran, and other countries (Bové et al., 2000; Chung, Khan & Brlansky, 2006; Al-Ghaithi et al., 2016). However, our previous findings showed that symptom expression due to WBD is not apparent in some areas, especially deserts and monsoon areas (Al-Ghaithi et al., 2017). Although it was clear that there was a relationship between symptom expression and geography, it was questioned whether phytoplasma infecting trees expressing WBD symptoms could be the same as phytoplasmas from trees not expressing WBD symptoms.

Phylogenetic analysis showed that all phytoplasma isolates share the same sequences of the *imp* and *SAP11* genes. This resulted in a lack of clustering of phytoplasma isolates from different climatic conditions, showing that phytoplasma from symptomatic and asymptomatic trees have the same *imp* and *SAP11* gene sequence.

Previous studies using the 16S rRNA gene showed limited variation between phytoplasmas which belong to the same group (Bertaccini & Duduk, 2009). Similarly, low variation was detected between different isolates of “*Ca.* P. aurantifolia” from three different countries (Oman, UAE, and Iran) based on sequences of the 16s rRNA, *secA*, and *imp* genes. These three genes could not separate strains based on the country from which they were obtained. Findings from our study were in agreement with Al-Abadi et al. (2016) who showed limited variation in the *imp* gene.

Although the SAP11 gene is associated with symptom induction (Sugio et al., 2011), no differences were found in the sequence of this gene between isolates obtained from areas with or without WBD expression. Thus, this result and the above results confirm that all phytoplasma isolates are identical and have low genetic diversity. They do not support the possible presence of different phytoplasma strains in the studied locations and trees.

## Conclusion

This study shows that WBD phytoplasma from semitropical areas, subtropical areas, and desert areas share a very high level of genetic similarity based on imp and SAP11 genes. This gives indication that acid lime trees in these locations are affected by the same phytoplasma strain, but symptom development is affected by environmental factors rather than by phytoplasma strains. Also, symptom development can be affected by other parameters such as soil moisture or/and plant cultivars or/and cultural practices or even coinfection with other pathogens/strains. Future studies should address the relationship between symptom expression in acid lime and other possible factors.

## Additional Information and Declarations

### Field Study Permissions

The following information was supplied relating to field study approvals (i.e., approving body and any reference numbers): Permits are not required for the collection of samples from Oman.

### Data Availability

The following information was supplied regarding data availability: All GenBank accession numbers are provided in Table 2.

## References

[ref-1] Al-Abadi SY, Al-Sadi AM, Dickinson M, Al-Hammadi MS, Al-Shariqi R, Al-Yahyai RA, Kazerooni EA, Bertaccini A (2016). Population genetic analysis reveals a low level of genetic diversity of ‘*Candidatus* Phytoplasma aurantifolia’ causing witches’ broom disease in lime. SpringerPlus.

[ref-2] Al-Ghaithi AG, Al-Sadi AM, Al-Hammadi MS, Al-Shariqi RM, Al-Yahyai RA, Al-Mahmooli IH, Carvalho CM, Elliot SL, Hogenhout S (2017). Expression of phytoplasma-induced witches’ broom disease symptoms in acid lime (*Citrus aurantifolia*) trees is affected by climatic conditions. Plant Pathology.

[ref-3] Al-Ghaithi AG, Hanif MA, Al-Busaidi WM, Al-Sadi AM (2016). Increased sodium and fluctuations in minerals in acid limes expressing witches’ broom symptoms. SpringerPlus.

[ref-4] Al-Sadi AM, Al-Moqbali HS, Al-Yahyai RA, Al-Said FA (2012). AFLP data suggest a potential role for the low genetic diversity of acid lime (*Citrus aurantifolia* Swingle) in Oman in the outbreak of witches’ broom disease of lime. Euphytica.

[ref-5] Al-Sakeiti MA, Al-Subhi AM, Al-Saady NA, Deadman ML (2005). First report of witches’-broom disease of sesame (*Sesamum indicum*) in Oman. Plant Disease.

[ref-6] Al-Subhi AM, Hogenhout SA, Al-Yahyai RA, Al-Sadi AM (2017). Classification of a new phytoplasma subgroup 16SrII-W associated with Crotalaria witches’ broom diseases in Oman based on multigene sequence analysis. BMC Microbiology.

[ref-7] Al-Yahyai RA, Al-Sadi AM, Al-Said FAJ, Alkalbani ZH, Carvalho CM, Elliot SL, Bertaccini A (2015). Development and morphological changes in leaves and branches of acid lime (*Citrus aurantifolia*) affected by witches’ broom. Phytopathologia Mediterranea.

[ref-8] Aryamanesh N, Al-Subhi AM, Snowball R, Yan G, Siddique KHM (2011). First report of Bituminaria Witches’-broom in Australia caused by a 16SrII phytoplasma. Plant Disease.

[ref-9] Bai X, Correa VR, Toruño TY, Ammar E-D, Kamoun S, Hogenhout SA (2009). AY-WB phytoplasma secretes a protein that targets plant cell nuclei. Molecular Plant-Microbe Interactions.

[ref-10] Bekele B, Abeysinghe S, Hoat TX, Hodgetts J, Dickinson M (2011). Development of specific secA-based diagnostics for the 16SrXI and 16SrXIV phytoplasmas of the Gramineae. Bulletin of Insectology.

[ref-11] Bertaccini A, Duduk B (2009). Phytoplasma and phytoplasma diseases: a review of recent research. Phytopathologia Mediterranea.

[ref-12] Bové JM, Danet JL, Bananej K, Hassanzadeh N, Taghizadeh M, Salehi M, Garnier M (2000). Witches’ broom disease of lime (WBDL) in Iran.

[ref-13] Chung KR, Khan IA, Brlansky RH (2006). Citrus diseases exotic to Florida: witches’ broom disease of lime (WBDL). EDIS Publications, University of Florida.

[ref-14] Contaldo N, Bertaccini A, Paltrinieri S, Windsor HM, David Windsor G (2012). Axenic culture of plant pathogenic phytoplasmas. Phytopathologia Mediterranea.

[ref-15] Contaldo N, Satta E, Zambon Y, Paltrinieri S, Bertaccini A (2016). Development and evaluation of different complex media for phytoplasma isolation and growth. Journal of Microbiological Methods.

[ref-16] Davino S, Calari A, Davino M, Tessitori M, Bertaccini A, Bellardi MG (2007). Virescence of tenweeks stock associated to phytoplasma infection in Sicily. Bulletin of Insectology.

[ref-17] Dickinson M, Hodgetts J (2013). Phytoplasma Methods and Protocols.

[ref-18] Doyle JJ, Doyle JL (1987). A rapid DNA isolation procedure for small amount of fresh leaf tissue. Phytochemcial Bulletin.

[ref-19] Frost KE, Esker PD, Van Haren R, Kotolski L, Groves RL (2013). Seasonal patterns of aster leafhopper (Hemiptera: *Cicadellidae*) abundance and aster yellows phytoplasma infectivity in Wisconsin carrot fields. Environmental Entomology.

[ref-20] Harling R, Arocha Y, Harju V, Tobing C, Boa E, Kelly P, Reeder R (2009). First report of 16SrII ‘*Candidatus* Phytoplasma aurantifolia’ infecting chilli and tamarillo in Indonesia. Plant Pathology.

[ref-21] Hernandez-Perez R, Noa-Carrazana JC, Gaspar R, Mata P, Flores-Estevez N (2009). Detection of phytoplasma on indian fig (*Opuntia ficus-indica* Mill) in Mexico Central Region. OnLine Journal of Biological Sciences.

[ref-22] Hogenhout SA, Oshima K, Ammar ED, Kakizawa S, Kingdom HN, Namba S (2008). Phytoplasmas: bacteria that manipulate plants and insects. Molecular Plant Pathology.

[ref-23] Khan AJ, Botti S, Al-Subhi AM, Gundersen-Rindal DE, Bertaccini AF (2002). Molecular identification of a new phytoplasma associated with alfalfa witches’-broom in Oman. Phytopathology.

[ref-24] Mehdi A, Baranwal VK, Kochu Babu M, Praveena D (2012). Sequence analysis of 16S rRNA and secA genes confirms the association of 16SrI-B subgroup phytoplasma with oil palm (*Elaeis guineensis* Jacq.) stunting disease in India. Journal of Phytopathology.

[ref-25] Parrella G, Paltrinieri S, Botti S, Bertaccini A (2008). Molecular identification of phytoplasmas from virescent Ranunculus plants and from leafhoppers in Southern Italian crops. Journal of Plant Pathology.

[ref-26] Queiroz RB, Donkersley P, Silva FN, Al-Mahmmoli IH, Al-Sadi AM, Carvalho CM, Elliot SL (2016). Invasive mutualisms between a plant pathogen and insect vectors in the Middle East and Brazil. Royal Society Open Science.

[ref-27] Rashidi M, D’Amelio R, Galetto L, Marzachì C, Bosco D (2014). Interactive transmission of two phytoplasmas by the vector insect. Annals of Applied Biology.

[ref-28] Siampour M, Izadpanah K, Galetto L, Salehi M, Marzachí C (2012). Molecular characterization, phylogenetic comparison and serological relationship of the IMP protein of several ‘*Candidatus* Phytoplasma aurantifolia’ strains. Plant Pathology.

[ref-29] Sugio A, Kingdom HN, MacLean AM, Grieve VM, Hogenhout SA (2011). Phytoplasma protein effector SAP11 enhances insect vector reproduction by manipulating plant development and defense hormone biosynthesis. Proceedings of the National Academy of Sciences of the United States of America.

[ref-30] Tamura K, Stecher G, Peterson D, Filipski A, Kumar S (2013). MEGA6: molecular evolutionary genetics analysis version 6.0. Molecular Biology and Evolution.

[ref-31] Tolu G, Botti S, Garau R, Prota VA, Sechi A, Prota U, Bertaccni A (2006). Identification of a 16SrII-E phytoplasma in *Calendula arvensis, Solanum nigrum, and Chenopodium* spp. Plant Disease.

[ref-32] Weintraub P, Jones P (2010). Phytoplasmas: Genomes, Plant Hosts and Vectors.

[ref-33] Zamora L, Acosta K, Martínez Y (2012). First report of ‘*Candidatus* Phytoplasma asteris’ (16SrI group) affecting common bean in Cuba. New Disease Reports.

